# How to Intervene in the Health Management of the Oncological Patient and of Their Caregiver? A Narrative Review in the Psycho-Oncology Field

**DOI:** 10.3390/bs11070099

**Published:** 2021-07-09

**Authors:** Gian Piero Turchi, Marta Silvia Dalla Riva, Luisa Orrù, Eleonora Pinto

**Affiliations:** 1 Department of Philosophy, Sociology, Pedagogy and Applied Psychology, University of Padua, 35131 Padua, Italy; gianpiero.turchi@unipd.it (G.P.T.); martasilvia.dallariva@unipd.it (M.S.D.R.); luisa.orru@unipd.it (L.O.); 2 Unit of Surgical Oncology of the Esophagus and Digestive Tract, Veneto Institute of Oncology, 35128 Padua, Italy

**Keywords:** oncology, health, caregiver, psycho-oncology, psychological support, healthcare, quality of life, health management, cancer, community

## Abstract

Starting from statistical data derived from the oncological field, some articles have highlighted the importance of communication in the patient–caregiver dyad and have considered the various roles involved in a cancer diagnosis situation. Thus, the question of how to intervene in terms of “quality of life” from the time of diagnosis to the recovery or death of a cancer patient, beyond the sanitary and physical dimensions, has become relevant. Therefore, the present narrative review aims to offer an overview of the state of the art in terms of the psychological treatment modalities of cancer patients, from the diagnosis to the post-surgery period. A total of 67 articles were collected and analyzed, in relation to (1) psychological constructs employed in the oncological field, (2) intervention models and (3) quality of life and well-being measurement and evaluation tools. We described these articles, differentiating between those focusing on the role of (1) the patient, (2) the caregiver, (3) the patient–caregiver dyad and (4) healthcare professional roles. The oncological diagnosis and its repercussions in the lives of the patient and caregiver were explored and critical aspects that emerged from the literature were highlighted. In conclusion, the analysis allowed some considerations about the need to define research protocols and useful management strategies for increasing the overall health of patients with cancer diagnoses and the people who surround them.

## 1. Introduction

Recently, the data related to the amount of cancer diagnoses have shown several changes. The yearly report by the American Cancer Society (ACS) and the International Agency for Research on Cancer (IARC) [1] indicates that in 2020 the number of new cancer cases was about 19.3 million, mainly related to breast cancer (11.7% of new diagnoses) and lung cancer (11.4%). Moreover, it is foreseen that in 2040 the figure will reach 28.4 million cancer cases, with an increase of 47% against 2020. The deaths total up to about 10 million, with, in the first place, lung cancer (18%), followed by colon cancer (9.4%) and liver cancer (8.3%). The report highlights, as well, an exponential increase, in developing countries, of cancer cases that are relatively simple to prevent and treat in developed countries (such as cervix or breast neoplasm). Against these data, the report authors indicate the lack of cancer prevention measures and of facilities for cancer treatment in the developing countries, which could create a future overload for the national health care systems in terms of the management of cancer patients. 

The report adopts statistical data that are mainly related to health care, thus linked to the physical body, to offer in anticipation of the above mentioned critical aspects becoming crucial in the future. Then, the need to consider a global health dimension of the individuals that suffer from cancer is inferred, beyond the body dimension. This kind of consideration emerged from a literature analysis: the management of the purely medical plan in patients diagnosed with cancer is not sufficient to cover the needs that the patient expresses on a psychological and interactive level [2]. Focusing solely on medical terms runs the risk of leaving to random chance all those aspects that play a part in constructing the reality of an “oncological diagnosis”, in which various roles, in interaction with each other, contribute to its generation and management. Therefore, asking questions that are directly aimed at constructing management solutions just for patient’s wellbeing, does not allow for an effective taking charge [2,3]. Conversely, the global consideration of underlying needs in patients’ daily lives (patients who are biographically and necessarily in relation to themselves, to others, to the contextual elements in which they are embedded) allows the contemplation of all those aspects and roles involved in patients’ configuration of reality [4] after cancer diagnosis [5], as well as all the narrations about “being a cancer patient” [6]. Therefore, what are the key points defining useful aspects for the management of the patient with cancer diagnosis as a whole? Answering this question could be possible by starting from the basic elements of an oncological configuration and the possibility of seeing the cancer patient as part of an interactive network. In this interactive network there are also caregivers, families, friends, healthcare professionals, and facilities. Furthermore, this network can actively take charge of the medical situation and its interactive repercussions [2], intercepting the critical situation without merely waiting for a solution to arise [7].

Therefore, how does the patient manage the physical illness in the management plan of his own life, in the light of the same diagnosis? How does the diagnosis have an impact on the various aspects of the patient’s life? How is the diagnosis itself managed by the caregivers surrounding and caring for the affected individual? Against the diagnosis, what is the role covered by the health professionals in the management of everything that is not related to the body, but rather, relates to the interaction between the patient and that which surrounds him/her? These questions have been formulated in a manner consistent with the above. They became useful for the identification of the main research question, that is **how** **to intervene in terms of “quality of life” from the time of diagnosis to the recovery or death of a cancer patient** (that is, in the cases where the prevention processes have failed and where it is necessary to take charge of the patient at 360°, not only in physical terms, but also at the level of interaction)?

This contribution aims to offer an overview of the state of the art in terms of the psychological treatment modalities of cancer patients, and their caregivers, as the role that continuously interact with them, from the time of diagnosis to the post-surgery period. The considerations in this paper go beyond the physical management of a cancer patient: the caregiver role can be considered as a hub that is influenced by the repercussions of diagnosis and as one that offers a contribution to the management of illness beyond the health dimension, also taking into account the working and everyday activity dimensions. The caregiver plays, in fact, an important role in the management of the patient and of their life, thanks to the support that he/she is able to offer at the level of interaction, not only at the physical level.

The narrative review will cover the constructs, intervention methods, and tools for measuring and evaluating psychological constructs, employed in recent years, at the psycho-oncological level, taking into account both the patient and the caregiver. Moreover, it will offer insights into the repercussions of illness management, considering the role covered by health professionals.

## 2. Materials and Methods

The literature analysis has been carried out starting from the material collection within the PubMed, Scopus and Google Scholar platforms to identify recent research related to the health management of oncological patients and of their caregivers. The search for the material started from some contributions that highlighted the importance of communication in the patient–caregiver dyad [8,9,10]: these generated questions in the researchers, about how it is currently intervened within the dyad to promote an effective management of the repercussions of oncological diagnosis, beyond the purely medical management of the diagnosis itself. According to the focus of the research, three points were considered: (1) psychological constructs employed at cancer level, (2) intervention models and (3) evaluation tools used for cancer patient Quality of Life and well-being assessment. These three subjects have been mixed with the involved roles in cancer diagnosis situations (patients, caregivers, health care professionals), deepening the diagnoses’ repercussions in patients’ life, focusing in particular on the working and daily activities dimension.

In this research, articles were collected in April 2021 through the following search keywords, which gave a total of 92 articles (33 in Pubmed, 38 in Scopus, 21 in Google Scholar):

1. Oncology and caregivers; oncology and workers; oncology and psychology and caregiver;
2. Oncology and treatment and workers; oncology and treatment and caregiver;
3. Oncology and care and workers; oncology and psychology and support.

Articles strictly considering health care terms and articles strictly proposing theoretical reflections on psychological constructs used in the field of oncology were excluded, since they did not regard the research focus—the management of the cancer patient and of their caregiver in terms of health and quality of life. So, 24 articles were excluded. Instead, the contributions of original research kind have been considered: quality, quantity and quality–quantity studies; essays involving several roles involved in the cancer diagnosis situation (patients, caregivers, professional roles); studies considering the various stages of the treatment path; essays considering several cancer diagnoses (kind of cancer and involved organs).

The considered contributions cover a period of about twelve years, between 2009 and 2021, while most of the sources are from between 2016 and 2021. We considered also two articles dating back to the 1990s, to provide a foundation to two of the measurement tools.

In total, then, 67 papers were kept. The analysis of the sources can be found in the table describing the sources, which is attached to this contribution as supplementary material.

## 3. Psychological Support in the Oncological Field: Patient and Caregiver

Against the reading of the collected material, it has been possible to organize the sources subdividing them into three content macro categories. The first category focuses on the constructs whose study object is the psycho-oncological one (for instance: emotions, stress, depression, but also decision-making and needs). The second category focuses on the recent developments offered by the literature regarding the intervention models and programs regarding the psycho-oncological field. Lastly, the third category considers the measurement and evaluation tools validated by the literature in recent years.

In general terms, the literature has oriented itself more and more towards offering useful solutions to promote a good quality of life in the roles involved at oncological level (patients, caregivers, health care professionals): the various constructs, tools and intervention methods are linked to the wider dimensions of mental health (MH) wellness and quality of life (QoL), giving emphasis also to the caregiver role and to their life quality inside the relationship with the patient, often compromised of the period after diagnosis [11,12].

Below, we offer a detailed overview of what emerged in the three content categories, differentiating between (1) patient; (2) caregiver; (3) patient–caregiver dyad and, where possible, (4) health-care professional roles.

### 3.1. Psycho-Oncological Constructs Investigated in Roles Involved in Cancer Diagnosis Situations

#### 3.1.1. Patients

Patients constitute the first category to be discussed. Several contributions have deepened the stress dimension in cancer patients [12,13,14]. To this purpose, Granek and colleagues have promoted an innovative investigation modality [15], deepening the stress and depression constructs in cancer patients starting from the health workers point of view (oncology health care professionals, OHCp), investigating through the Grounded Theory (GT) the potential distress causes in the patients, perceived by the professionals. Among these causes, the health care professionals state three content macro categories: factors linked to the illness (side effects of the disorder and treatment, loss of physical functions, worrying about the body image); social factors (socio-economical stress, loneliness and lack of social support, stress linked to the family); existential factors (addiction/fear of being a burden, death anxiety, loss of meaning). Overall, the authors report that, in the psycho-oncological field, some contents emerged from the investigation, in particular, the socio-economical stress and the stress linked to the family dimension have not been deepened in the literature yet, in their link with life quality.

Other constructs that emerge from the studies in the literature are those of “choice” in terms of deciding about the treatment to be undertaken and the use of a psychological support service, as well as motivations behind the choice. Hannon and colleagues [16], through a qualitative investigation based on the Grounded Theory (GT), have shown the importance of freedom of choice of the treatment chosen by the patients, who consider as fundamental the availability of information and confrontation moments with the professional roles that be of support in the decision about the treatment to be taken. The choice construct is considered also by Ann-Yi and colleagues [17], who direct their double-blind randomized cross-over trial towards the understanding of how the psychological support service introduction modalities to the cancer patients can influence their choice to rely on the service. The participants do not report preferences about the psychological support introduction modalities, i.e., the choice to use the service does not depend on the role introducing it (oncologist vs. counsellor).

Additionally, Isaksson and colleagues have directed themselves towards the study of the motivations at the base of the patients search for psychological support: the results of the study stress how the patients look for this professional support in order to overcome the critical oncological situation and manage the relations and daily life [18]. Washington and colleagues [19] aim to investigate the factors influencing the involvement in the participation to support groups by caregivers: the researchers have shown how (1) emotional isolation and inactivity periods, (2) contents related to the death and pain subjects and (3) meetings discreet delivery modes and guaranteeing privacy enhance the caregivers’ motivation to join support groups services. Such aspects, though, do not ensure that caregivers adopt active modalities in joining the meetings.

In 2019, Aert and colleagues studied psychological constructs linked to the role of oncological patients [20]: through linear regression models, they investigated the relation between the emotional regulation strategies and the cognitive functioning and the emotional wellness. This study shows how the emotional reappraisal is a useful adaptive strategy of emotional regulation, helping the patients to experience less anxiety and worrying after intervention, against the patients that explicitly express their emotions.

#### 3.1.2. Caregivers

Furthermore, other research has focused on the study of the stress, depression and anxiety constructs of caregivers, such as Barrera and colleagues, who have investigated the anxiety levels of brothers and sisters caring for siblings suffering from cancer [21]. Heckel and colleagues [22] have examined the relation between depression level and unsatisfied needs among the caregivers of cancer patients of recent diagnosis: 57% of the caregivers involved in the study report at least one unsatisfied need, mainly within the information and treatment services need topic. Moreover, one third of caregivers shows high depression levels against the administration of the Centre for Epidemiologic Studies-Depression Scale (CES-D), and the analysis of the collected data indicates a correlation between the high number of unsatisfied needs and depression symptoms.

#### 3.1.3. Patient–Caregiver Dyad

Against the need to deepen the changes in terms of stress for the patient–caregiver dyad, thus in interaction between them, Douglas and colleagues [23] have conducted a study aimed at putting into relation the quality of life linked to physical and psychological health of the patients with the psychological and emotional state of the caregiver, a span covering the various illness management stages, from diagnosis to post-intervention. The influence of the patient and caregiver’s emotional states emerge from the results as moderately correlated and stable over the course of time: this study has highlighted the need to intervene at the same time and in an integrated way for both the patient and the caregiver.

This overview about the analyzed constructs in the literature included a study by Johansen and colleagues [24]. Authors have dealt with the investigation of the patient–caregiver dyad through the administration of tools such as the Cancer Behavior Inventory (CBI), the Caregiver Reaction Assessment (CRA), measurements scales of some constructs (the General Sleep Disturbance Scale, the Center for Epidemiologic Studies Depression Scale, the Medical Outcomes Study Social Support Survey) and questionnaires concerning the collection of demographic information: the linear regression analysis adopted has made emerge a significant link between the caregiver burden and the self-efficacy variables, sleep disorders and social support linked to the patient (considered in the variables of emotional/informational, instrumental and affective support and positive social interactions—dimensions assessed by the 20-item Medical Outcomes Study Social Support Survey). Among the variables linked to the caregiver that influence the caregiver burden perception, the data analysis has stressed a significant link between high depression, tiredness, and depressive symptoms scores, above all in women. The authors concluded that, since the beginning of treatment, the burden perceived by the caregiver is influenced by the interdependence between the patient characteristics and their depressive symptoms and problems. Additionally, in this study, as in the previous ones, the authors stress the importance of deepening how patients and caregivers influence each other, and the repercussions of that in illness management.

### 3.2. Methods of Supportive Intervention for Patients, Caregivers and Health Care Roles Involved in Cancer Diagnosis Settings

#### 3.2.1. Targeted at the Patient–Caregiver Dyad

The last year and a half, characterized by the pandemic event that struck the world, has made it necessary to develop new management modalities of psychological support interventions in all the fields where they are employed, including the oncological field. As for, then, the second macro category of investigation content, the need to deepen online interventions and programs aimed at supporting oncological patients and caregivers emerged from several researchers, among which are Washington and colleagues [19]. These modalities allow to overcome the “participation barriers” against the support and reciprocal help groups that are carried out in person (face-to-face). In fact, the closed group social platforms also allow to guarantee a certain privacy level, though they do not offer necessarily a high level of effectiveness in the dimension of the commitment of those joining the groups [19].

Another example of study that investigate the possibility of online intervention programs for patient–caregiver dyads is the one of Lambert and colleagues [25]: the study presents an innovative intervention method, called TEMPO (Tailored, wEb-based, psychosocial and physical activity (PA) self-Management PrOgram), designed to support oncological dyads in their psycho-social needs, by building strategies for the autonomous management of physical, psychological and relational difficulties. Starting from a need assessment, the 10-week online program focuses on setting goals and plans for the future, considering moments of progress monitoring and strengths evaluation. The program allows patients and caregivers to take back their choices, considering the presence of other people around them.

#### 3.2.2. Targeted at Caregivers

Another kind of remote intervention has been validated by Heckel and colleagues [26] and by other scholars [27,28]: the “13 11 20 outcall program” offers the caregivers the chance of telephone interfacing with health care professionals trained to manage the questions, doubts and issues brought by the users, directing them towards useful services and monitoring the support path trend. This kind of service has proved with time to lower the stress level of the caregivers contacted. The most frequent topic in the phone calls has been categorized under the “psychological distress” label.

Others, like Hendrix and colleagues, have found the need to develop caregiver’s self-efficacy and management strategies of the stress experienced by their relatives with oncological diagnosis [29]. The latter study has brought to light how training is effective in the perception of self-efficacy of caregivers, but less in the management of stress experienced by the caregiver in first person. Again, as for the training strategy, the literature also proposes the Life Review Therapy (LRT) and the Memory Specificity Training (MST) [30]. LRT (in individual or group sessions), conceived by psychiatrist Dr. Robert Butler, is based on the attribution of value to past life events (concerning various areas of reflection, such as education, health, relevant events, etc.), to increase the level of personal empowerment in managing the future and to decrease the impact of depressive symptoms [31]. The cognitivist-based MEST also considers autobiographical memory to increase its specificity, with effects on depressive symptoms related to traumatic events [32,33]. Both of the above-mentioned interventions have been transferred and adapted to the oncological setting, as the roles involved in this setting can experience depressive symptoms with repercussions on overall well-being and quality of life [29].

#### 3.2.3. Targeted at Patients

Among the online support interventions, Lozano-Lozano and colleagues [34] have validated the application BENECA mHealth, used in parallel with a supervised rehabilitation program, highlighting its efficacy on the life quality increase in people who survived breast cancer. The application allows to offer the oncological patients, who survived cancer, tips on nutrition and physical exercise, needed to avoid illness repercussions and life quality level decrease [34]. Another integrated support program validated by Jenniches and colleagues is called “integrated cross-sectoral psycho-oncology program” (isPo): this reconciles several kinds of treatment and support, from the “cancer self-help”, to the “psychosocial cancer counselling”, to the “psycho-oncological psychotherapy”, integrated in a coherent way with the oncological health care treatment programs. The study, as for the program in question, offers, then, the bases on which decisions can be taken against the chance to integrate the psyco-oncological dimension directed towards the need in the patients’ treatment paths [35]. In the article’s conclusions, authors highlight the possibility of integrating this kind of care system with palliative care and other approaches of disease management, such as music and art-therapy.

Actually, moving to the end-of-life oncological patients, two studies by Johnson and colleagues propose interventions of Advanced care planning (ACP) [36,37] and validate its efficacy: starting from poor participation to discussions on end-of-life by patients, ACP allows to support them in the understanding of their needs (values, desires, physical needs, preferences, future perspectives) related to the treatment path. Putting this kind of program aside the standard health care treatment allows to increase the quality level of life and death in this target and diminish the stress level perceived by the caregiver in health care environments where is lacking the communication among patients, relatives, and health care professionals, but not in environments where such communication is already found. The study allows to bring to light the need for interactive and user base management skills to be promoted among health care professionals. Thus, ACP programs become useful in environments where a need to work on the interaction between the doctor and oncological patients is found. 

At the same time, researchers have moved on the role of early palliative care [16] and integrated palliative care [38], and on their positive influence on the increase in the coping strategies of the diagnosis management and treatment path, as well as on the quality of life [39].

#### 3.2.4. For Health-Care Professional Roles

In some cases, the support interventions are performed as a training, in particular for the medical and health care roles and social-workers (Oncology Social Workers—OSW). Quillen and colleagues, in fact, have highlighted how the health care professional roles report to feel themselves competent and comfortable in the communication support and related to the illness management, while they feel less skilled to deal with discussions about end-of-life. The same roles ask more information about this aspect [40] and to be involved in the offer of support to the caregivers to diminish their stress level [26]. Several researchers have in fact dealt with this aspect: Aubin and colleagues have decided to intervene on the development of competences in the nursing roles [13]. Health care professionals are deemed to be a key role in supporting the caregivers of oncological patients [14,24,29], though the study in this respect reports low levels of trust and low knowledge of the intervention modalities by the same roles toward the caregivers, levels that however can be increased whenever it has been undertaken the caregiver position or the one of oncological patient in life [41].

### 3.3. The QoL and Well-Being Evaluation Tools

As for the third macro category of investigation, at the state of the art, several evaluation tools have been validated in the psycho-oncological field. Among those of quality of life (QoL) assessments, the instruments offered and validated by the European Organization for Research and Treatment of Cancer stand out [42]: the Quality of Life of Cancer Patient in the extended version (QLQ-C30) and in diseases specific versions or concerning specific conditions, for instance patients treated through palliative care (QLQ-C15-PAL). The EORTC also offers a tool to measure patient satisfaction with the care they receive (Satisfaction with in-patient cancer care; IN-PATSAT32). Another tool used in the oncology field for measuring health-related quality of life is the EQ-5D, both in the EQ-5D-3L and EQ-5D -5L versions [43]. 

Going deeper into what is available in the literature, one of the most frequent tools is also the Caregiver Quality of Life Index-Cancer (CQOLC-K), conceived and validated in 1999 by Weitzner and colleagues [44]. In the last ten years, several researchers have employed this tool within studies allowing its validation in its different cultural versions, allowing the use of these tools in different countries. This will help to address the issues raised in the IARC report [1] regarding differences in cancer prevention measures between countries around the world. Among the different cultural versions of CqoLC-K: the Turkish one, validated by Yakar and Pinar in 2013 [45]; the Japanese one, adopted by Sugyiama and colleagues [46]; the Korean one, validated by Ando and colleagues in 2013 [47] and used within a validation study for a wider measurement tool of the quality of life in patients with prostatic cancer (Expanded Prostate Cancer Index Composite—EPIC [48]) and in a study with which some researchers study how the oncological patients esteem being a burden for one’s own caregivers and how this is linked to the self-evaluation of the latter against the life quality and the anxiety and depression levels measured through the Hospital Anxiety and Depression Scale [12].

The authors of the last above-mentioned research in 2014 have validated the Korean version of the Cancer Communication Assessment Tool (CCAT-PF [49]) in over 990 patient–caregiver dyads, showing how this can be overlapped in psychometric terms to the original English version conceived by Siminoff and colleagues in 2008 [50]. The tool allows to find the coherence level of the communication modalities between patient and caregiver.

Another widely used tool in oncological environments is the Distress Thermometer, which is used in the measurement of stress levels of those involved in oncology-related environments, above all caregivers [24] and those in health care and social and health care roles [51].

Cella and colleagues, in 1993, have validated a further evaluation tool, the Functional Assessment of Cancer Therapy–General (FACT-G) questionnaire [52]: its complete version (38 items) allows to measure various constructs of the quality of life, such as the physical wellbeing, the functional one, and the social and emotional ones. This tool is used largely in oncological contexts thanks to its speed and ease of use, reliability, validity, and reactivity to changes. It has been employed by Greer and colleagues in a study that proved that the early and integrated palliative care (EIPC) increases the life quality level of oncological patients through the mediation of approach-oriented coping strategies [39]. The literature also makes available the seven-item version, validated by Mah and colleagues last year [53], contemplating the physical and functional wellbeing dimensions.

## 4. Oncological Diagnosis Repercussions

In this contribution, the literature focusing on the interaction between patient and caregiver has been analyzed. The considered articles answered the research questions concerning how the patient manages their physical illness in the management plan of their own life in light of the same diagnosis, how the diagnosis itself is managed by the caregivers caring for the patients, which intervention methods are available and which tools are used in the psycho-oncological field concerning wellbeing. The effect of cancer diagnosis and the consequent treatment pathway on patients and caregivers’ lives, and the impact on everyday life, was also considered. 

So, to this purpose, another part of the scientific literature dedicated itself to the deepening of which repercussions the oncological diagnosis has on the various life scopes of the patient, in particular in the economical, working, family and daily life.

Starting from the economical point of view, a treatment path for the oncological patients involves management expenses. Necessarily, then, the economic-financial state of the patients and the expenses that they can afford has an influence on the treatment path of the oncological diagnosis and on the management choices they make [54]. The study by Boele and colleagues has highlighted the correlation between management costs and depression symptoms in patients, and between loss of productivity and fatigue in their caregivers [55]: in general terms, the authors evidence a strong link between management costs and treatable psychological states (depression, fatigue, cognitive commitment), indicating that an adequate psychological support for patients and caregivers can reduce the diagnosis management costs [56].

Furthermore, again, focusing on the occupational point of view, Short and colleagues, in two studies dating back to the first decade of 2000 [56,57], have stressed the influence of diagnosis on the long-term occupational state of patients. In this respect, this year has been brought to light the need of support paths for the post-surgery job reintegration [58,59]. However, the diagnosis repercussions within the work field literature lacks further scientific studies.

As for the management sphere of the care path of oncological patients, considering the family dimension, the literature brings to light how the patients tend to delegate to caregivers the management of the path, feeling overwhelmed by information [5] and by the complexity of the healthcare system [16]. In addition to that, Shin and colleagues point out that patients underestimate the problems undertaken by caregivers (in terms of quality of life, anxiety and depression) managing the situation, and how this can get worse with the decrease of the intra-family dialogue related to it [11].

Taking into account the care path management in daily life, Hall and colleagues have made a quality study, through the administration on semi-structured interviews, investigating the perception of time spent in oncological care by the patients, caregivers and oncology professionals. Given the uncertainty of the effectiveness of health care treatments, patients and caregivers report they dedicate their “chronological” time (that differs from the one labelled as “existential”) mainly to the cancer health care, reducing the chances to dedicate oneself to any other aspect of daily life [60]. Additionally, Hwang and colleagues [61] investigated how the cancer diagnosis (the role of symptoms as fatigue, in particular) affects patients’ ability to conduct everyday life activities: authors highlight how clinical relevant fatigue (CRF) decreases the QoL level in stomach cancer survivor’s patients.

Concluding the overview of the cancer diagnosis repercussions, resuming what was stated in Section 1 [1], even the health care system on the whole has to necessarily manage cancer diagnoses in terms of efficiency, considering the upward trend of cancer and survival cases (it is expected that these will increase from 16.9 million in 2019 to 22.1 million in 2030 [62]): in health care institutions, there are repercussions on the user base management, on the stress and burnout level of health care professionals [63], and regarding the spaces available. 

## 5. Discussion

Tracing the path since the beginning of this narrative review for its construction, contributions covering a time span of about twelve years between 2009 and 2020 were considered, with most sources concentrated between 2017 and 2020.

As a scoping review, this contribution aims at providing an overview of particular key aspects emerged in the last twelve years, useful to deepen the importance of considering the patient–caregiver dyad in interaction with other roles involved in oncological assets. This focus allows to highlight the benefits for healthcare systems and the community, informing us about the relative practices that should be implemented and the need for a comprehensive approach to what happens after a cancer diagnosis, in order to increase the effectiveness of interventions.

Sixty-eight articles were collected (in-depth study in Supplementary Materials, i.e., Studies Summary), organizing the sources into three macro-categories of content: constructs under study in psycho-oncology (e.g., emotions, stress, depression, but also decision-making and needs), models/programs of intervention in psycho-oncology and measurement and assessment tools in use. The narrative review therefore addresses the issues mentioned, specifically according to three dimensions: contributions that consider the patient, those that consider the caregiver, and those that consider the interaction of the two roles in the dyad. In addition, what is offered in the literature was considered regarding studies that focus on the role that healthcare professionals play in the management of cancer and its consequences. 

From the analysis of the literature, some strengths emerged that can contribute to the management of patients, caregivers and professional healthcare roles (as doctors, nurses, social workers).

For example, consulted studies highlight the health value that the community [2,64] can offer to the cancer patient [16,60]. Specifically, the interaction between patients and the designated caregivers has been taken into consideration, highlighting how the sharing of interactive processes in charge of individual roles can produce an increase in the effectiveness of the social-health intervention on the overall health of the patient [18]. In light of this, Hendrix and colleagues have identified the need to develop caregivers’ self-efficacy and coping strategies for dealing with the stress experienced by their loved ones with cancer diagnosis [29]. It has been shown that training can be an effective tool in promoting caregiver coping skills, but less so in managing the stress experienced by caregivers themselves.

In the papers analyzed, it is also emphasized that it is highly desirable to promote the use of tools which are heterogeneous in terms of type, constructs detected and effectiveness, and which facilitate communication and dialogue between the roles involved in the community in which the patient with cancer lives [8,9,10]. Again, compared to the interventions that emerged as prominent in the analyzed overview, in some cases the supporting ones are educational: Aubin and colleagues also chose to intervene on competence development for nursing roles [13].

What the literature highlights is therefore the need to offer support to patients and carers in terms of strategies to manage the oncological situation [5], which are as dedicated as possible to the specific configuration [23,65,66,67] and future-oriented [16], i.e., which allow the generation of possible but not yet verified future scenarios, concerning which the roles involved in oncological diagnosis settings can take decisions in advance.

It is also underlined the attention that the roles working for the health of the cancer patient serve, towards the cohesion of the family unit: it is specifically mentioned that the alignment of both roles (patient and caregiver) towards a common goal of health of the family unit impacts on the management of critical situations [11]. Researchers believe that this aspect could generate a change in the management of “emotions, stress, feelings”, which may occur during the cancer patient’s biographical path [23].

However, the consulted literature points out some critical aspects that emerged since the material collection. The overview of the literature in the oncological field described here highlights several gaps in the management of psychological support for patients and caregivers and in the role played by physicians, nurses and healthcare professionals.

First of all, a number of studies were found to be similar in terms of the constructs investigated and the results that emerged, with minor differences mostly related to what the same results allow to be added to what is known about the field, but not to how these same results can be used. There is a lack of thrust in the literature that offers operational tools useful for the management of the patient–caregiver dyad.

This is also true with regard to the role of health and social professionals: studies that consider these figures are rare and lack in offering useful elements to build ways to manage the oncological environment considering professional roles in interaction with patients and caregivers.

Actually, in healthcare roles, the authors add some critical aspects and offer considerations on the direction to take in order to manage the possibilities of overloading the national health care systems in advance [52,55], not only from the physical point of view, but also from the interactive one of global community health. 

Eventually, another critical aspect concerns the sharing meaning of “quality of life”, “wellbeing”, “health”: all the researchers consider in a different way these three concepts, compromising the replication of studies and the rigor of the used methodologies and methods. Criticality, this also has repercussions on the adequacy and effectiveness of managing strategies and intervention methods used, in our case, in oncological fields.

Taking charge of the various critical aspects outlined could increase the global health configuration of both the patient diagnosed with a cancer and the entire community in which they are inserted, with particular regard to the role of the caregiver.

## 6. Conclusions: Critical Aspects and Needs

From the literature analysis, a wide presence of studies and research in the psycho-oncological field has emerged, involving: oncological patients covering most of the diagnostic spectrum of this kind of illness; caregivers of oncological patients; health care professionals (nurses, oncologists, and social workers). The studies span from the deepening of psychological constructs to the validation of psychological evaluation tools, to the conceiving and validation of intervention programs useful for the management of the various roles involved in the oncological field. All the analyzed contributions refer to the wider dimension of the quality of life and the wellbeing of the latter, revealing the need for the health care system to integrate the body care with the care for anything that is generated by an oncological diagnosis in psychological and interactive terms.

To summarize, the state of the art of the literature examined shows the need to further define the strategies considered useful for increasing the overall health of patient with a cancer diagnosis, their effects and, last but not least, the evaluation of their effectiveness. The various studies analyzed, however, decline the construct of “health” in ways that are always different from one another: as emerged from the analysis of the literature, some consider stress, others’ emotions, others depressive symptoms, and still others communication between the members of the dyad. This variety of constructs is traced back to the broader dimension of health, but without ever offering a definition of it. It is therefore necessary to offer a common definition of health which allows to operate in a precise and shared way through the application of epistemologically founded measurement and evaluation tools, consistent with the definition of health that is chosen, as well as the provision of interventions to support the roles of the community involved in oncological diagnosis. 

It also seems clear that one should consider not only the utility that the roles contributing to the patient’s health offer, but also the health that they produce for themselves and for others, through a precise taking charge of all the roles with respect to the development of one’s own competences to cover the clinical, interactive, work and family plan.

Health care systems and the research field should consider the importance of healthcare personnel as figures who directly interact with the patient–caregiver dyads (and not only separately with them) and have an impact on their global health level: they could support the dyad from the moment of diagnosis onwards, using the caregiver as a resource in the management of the patient. In this sense, healthcare professionals can also be involved in the administration of measurement and evaluation tools and interventions for the dyad, monitoring the same: they could become an active part of the dyad’s management, not only from a bodily point of view, but especially in the interactive global health dimension.

In this way, the community could benefit from a network of services able to anticipate the critical effects of an oncological diagnosis, intervening in an effective and concerted manner with all available roles [7]. Hence, the management of patients with cancer diagnosis could concern not only patients or caregivers, but all the roles involved in the services network, citizens included [2,7].

In conclusion, from this review the need of constructing research and intervention protocols clearly emerges, in order to consider the discursive configuration of the “patient with neoplasms” as a whole, thus using all the voices composing such configuration: caregivers, health workers, family members, friends, work colleagues, etc., in interaction with each other [16]: actually, the literature does not consider the latter roles (friends, work colleagues, neighbors), who in any case can contribute, interactively speaking, to the health promotion of the roles directly involved in the consequences of cancer diagnosis.

## Supplementary Materials

The following are available online at https://www.mdpi.com/article/10.3390/bs11070099/s1, Table S1: Table. Studies Summary.
