# Supplementary material for: How to Intervene in the Health Management of the Oncological Patient and of Their Caregiver? A Narrative Review in the Psycho-Oncology Field
Source: Behav Sci (Basel). 2021 Jul 9;11(7):99. doi: 10.3390/bs11070099 (PMC8301046; doi:10.3390/bs11070099)
Supplement: Supplementary file 1 [file behavsci-11-00099-s001.zip › behavsci-1270403-supplementary.pdf]

Table S1. Table - Studies Summary

N°	Authors' name	Publication	Subjects	Measurements	Study design
1	Sung H, Ferlay J, Siegel, RL, et al	2020	185 countries	- Population-based cancer registries (PBCR)<br>- Incidence and mortality of cancer<br>- International Statistical Classification of Diseases and Related Health Problems 10th Revision (ICD-10)<br>- 4-tier Human Development Index - HDI according to a binary proxy of development	N/A
2	Turchi, GP, Dalla Riva, MS, Ciloni, C, et al.	2021	N/A	Social Cohesion: Social cohesion Index	N/A
3	Wadhwa, D, Burman, D, Swami, N, et al.	2011	191 caregivers - patients dyads	- Screening: Short Orientation–Memory–Concentration Test<br>- Caregiver QoL: Caregiver QOL Cancer scale (CQOLC)<br>- Caregiver Health and Functioning: Medical Outcomes Study Short Form<br>- HRQoL: Functional Assessment of Cancer Therapy General (FACT-G)<br>- Severity of Symptoms: Edmonton Symptom Assessment System<br>- Performance status: Eastern Cooperative Oncology Group (ECOG)<br>- Comorbidity: Charlson Comorbidity Index<br>- Questionnaire including care-related factors<br>- Demographic characteristics: demographic questionnaire	Cluster randomized trial<br><br>Statistical analyses: descriptive statistics; simple linear regression analysis for association between factors; backward stepwise multiple regression analysis
4	Pearce, S, Whelan, J, Kelly, D, et al.	2020	18 patients	- Story of participants: interviews<br>- Impact of illness and treatment: photography<br>- Thoughts and experience: handwritten research diary	Longitudinal narrative study (thematic maps, conceptual frameworks)
5	Cooley, ME, Nayak, MM, Abrahm, JL, et al	2017	64	Symptoms and quality of Life (SQL): focus groups	Qualitative approach (inductive content analysis)<br><br>Statistical analysis: descriptive statistic
6	Willig C	2011	N/A	- Role of diagnosis	Critical review

				- Role of discourse<br>- Discursive constructions of cancer and its meanings: cultural imperative to 'Think Positively'; cancer as a moral concern<br>- Phenomenological repercussions: engaging with suffering and mortality; managing responsibility<br>- Struggle for meaning: being constructed by and positioned within cancer discourse; social, historical, cultural context in which meaning is made	
7	Turchi, GP, Iudici, A, Faccio, E	2019	N/A	N/A	Guidelines for the generation of a community service for entrepreneurs
8	Badger, TA, Segrin, C, Figueredo, AJ, et al.	2010	71 patients and 70 caregivers	- Psychological well-being: Symptoms of depression:Center for Epidemiological Studies-Depression Scale (CES-D)<br>- Positive and negative affect: Positive and Negative Affect Schedule (PANAS)<br>- Stress: Perceived Stress Scale (PSS)<br>- Physical well-being :UCLA Prostate Cancer Index<br>- Fatigue: Multidimensional Fatigue Inventory (MFI)<br>- Prostate specific health-related quality of life: *ad hoc* index<br>- Social well-being: social well-being scale<br>- Social support from family: Perceived Social Support-Family scale (PSS-FA)<br>- Spiritual well-Being: Quality of Life-Breast Cancer questionnaire	Three-wave repeated measures experimental design<br><br>Statistical analyses: growth curve analyses (GCAs), by Multi-Level Modeling (MLM)
9	Lapid, MI, Atherton, PJ, Kung, S, et al	2016	131 caregiver	- Quality of Life: Caregiver Quality of Life Index-Cancer Scale (CQOLC); Linear Analogue Self-Assessment (LASA)<br>- Mood swings: Profile of Mood States-Brief (POMS-B)<br>- HRQoL: Functional Assessment of Cancer Therapy-General (FACT-G)	Randomized, controlled clinical trial<br><br>Statistical analysis: Wilcoxon procedures to comparison of scores with score change; two-tailed alternatives and a 5% type I error rate to test hypothesis; effect size ad indication of clinical meaning
10	Lee, KC, Yiin, JJ, Chao, YF.	2016	80 caregivers	- Subjective burden: Caregiver Reaction Assessment tool; Heart Rate Variability<br>- Objective burden: Symptom Distress Scale, Enforced Social Dependency Scale, amount of assistance provided in personal care,	Longitudinal repeated-measure design<br><br>Statistical analysis: descriptive analysis to identify differences between participants and

				homemaking, transportation, and health care; time spent in caregiving each day<br>- Coping strategies: Caregiver Self-efficacy Scale	non-participants; generalized estimating equations (GEEs)
11	Slev, VN, Mistiaen, P, Pasman, HR, et al	2016	10 reviews analysed	Categorized outcomes in 5 labels	Systematic meta-review
Psycho-oncological constructs investigated in roles involved in cancer diagnosis situation					
12	Shin, J, Lim, JW, Shin, D, et al	2018	990 patient-caregiver dyads	- Caregiver burden (CB): domains of burden from Caregiver Burden Inventory<br>- Patient-estimated caregiver burden (PECB): five-items author-created measure<br>- Family communication: Family Avoidance of Communication about Cancer (FACC) scale<br>- Reactions to caregiving: Korean version of the Caregiver Reaction Assessment (CRA-K)<br>- Caregivers quality of life: Korean version of the Caregiver Quality of Life Index-Cancer (CQOLC-K)<br>- Caregivers depression and anxiety: Korean version of the Hospital Anxiety and Depression Scale (HADS)<br>- Socio-demographic and medical characteristics: medical records	Cross-sectional survey<br><br>Statistical analyses: First, descriptive statistics; multivariable regression analysis
13	Aubin, M, Vézina, L, Verreault, R, et al.	2017	120 patient-caregiver dyads	- Medical characteristics: interviews<br>- Distress: Hospital Anxiety and Depression Scale (HADS)<br>- Prevalence of clinical distress: Psychological Distress Index used in the Quebec Health Survey (PDSQHS)<br>- Needs: Home Caregiver Need Survey<br>- Psychological burden: French version of the Caregiver Burden Scale in End of Life Care (CBS-EOLC)<br>- Preparedness for caregiving. Preparedness for Caregiving Scale<br>- Quality of Life: City of Hope-QoL Scale-Family Version<br>- Service and health care resource utilization	Two arm-randomized controlled trial<br><br>Statistical analyses: descriptive statistics; mixed models; analyses of variance
14	Blackstone, E, Lipson, AR, Douglas, SL Closer	2019	332 patient-distanc	- Demographic characteristics: Enrollment form<br>- Anxiety/depression: Patient-Reported Outcomes Measurement Information System (PROMIS®)	Randomized controlled trial

			e caregiver dyads	- Distress: National Comprehensive Cancer Network® (NCCN) Distress Thermometer<br>- Health status: Medical Outcomes Study-Short form<br>- Caregiver burden: Zarit burden interview-12<br>- Self-efficacy: Caregiving self-efficacy scale	Statistical analyses: descriptive analysis; covariance analysis; pearson correlation
15	Granek, L, Nakash, O, Ariad, S, et al	2019	61 oncology health care professionals	- Mental health distress and perceptions of causes of distress: semi structured interview guide	The grounded theory (GT) method (qualitative method); line-by-line coding and inductive, with codes and categories emerging from participants' narrative
16	Hannon, B, Swami, N, Rodin, G, et al	2016	26 patients<br>14 caregivers	Open-ended questions: quality of life, the quality of care provided, and how this had changed over the time period of the intervention, discussion of experiences with the palliative care team, benefit of intervention, participants' perceptions of the meaning of the term palliative care.	- Cluster-randomised controlled trial of early palliative care versus standard oncology care<br>- Qualitative grounded theory study using individual qualitative interviews
17	Ann-Yi, S, Tanco, K, Liu, DD, et al	2019	100 patients	- Demographics: questionnaire,<br>- Symptom distress scores: Edmonton Symptom Assessment System[ESAS],<br>- Current emotional distress: Hospital Anxiety and DepressionScale [HADS]<br>- previous experiences of psychiatric/psychology services other than from Supportive Care department: questionnaire<br>- General Attitudes Towards Psychology Service: 7-item questionnaire<br>- Barriers for psychology services: 1-item question	- double-blind randomized cross-over trial using Pocock-Simon randomization (factors: age, gender, race, time since diagnosi, HADS)<br>- 2 video vignettes: designed following Hillet and Van Vliet five recommended phases
18	Isaksson, J, Lilliehorn, S, Salander, P	2018	134 Oncology Social Workers (OSWs)<br>226 patient cases	- Present study focus: OSWs clinical duty<br>- Description of the first three patient cases that OSWs met face-to-face during the previous week: how the case was referred to them,what they perceived as the patient's initial motive for contacting them,additional motives that came up during the consultations, patient's age, gender, diagnosis, and an estimation of how many sessions the case would probably call for	- Part of larger project: nationwide survey conducted
19	Washington, KT, Oliver,	2019	58 caregivers	- Engagement (context, content, delivery): individual and group interviews.	Cluster crossover randomized pragmatic trial<br><br>- Secondary qualitative data analysis

	DP, Benson, JJ, et al				- Template analysis
20	Aerts, H, Van Vrekhem, T, Stas, L, et al	2019	- 28 Patients and 11 caregivers<br>-22 patients and 10 caregiver	- Cognitive reappraisal and expressive suppression: Emotion Regulation Questionnaire (ERQ)<br>- Use of processing and expression strategies: Coping through Emotion Approach scale (EAC)<br>- Cognitive performance: Cambridge Neuropsy-chological Test Automated Battery (CANTAB) in particular sustained attention (Rapid Visual Information Processing [RVP]), working memory (Spatial Span [SSP]), information processing speed (Reaction Time [RTI]), and executive functioning (Stockings of Cambridge[SOC])<br>- The extent to which people generally tend to worry: Penn State Worry Questionnaire (PSWQ)<br>- Emotional distress: State-Trait Anxiety Inventory (STAI-S)<br>- Loneliness: UCLA Loneliness Scale	Exploratory longitudinal study<br><br>- Statistical analysis: multivariate analysis of variance (MANOVA); linear regression; sequential Bonferroni correction
21	Barrera, M, Atenafu, EG, Schulte, F, et al	2018	75 healthy siblings of children on or off treatment	- Anxiety symptoms: Multidimensional Anxiety Scale for Children (MASC)<br>- Caregiver anxiety symptoms: Multidimensional Anxiety Questionnaire (MAQ)	Parallel group randomized controlled trial with two arms and three measurement time points<br><br>Statistical analyses: descriptive analysis; preliminary bivariate correlations, Chi-square test and ANOVA; multivariable mixed model analyses ; separate multivariable analysis
22	Heckel, L, Heckel, KM, Reynolds, J, et al	2015	150 patient-caregiver dyads	- Demographic characteristics: questionnaire<br>- Carer unmet needs: Supportive Care Needs Survey for Partners & Caregivers (SCNS-P&C)<br>- Depression: Centre for Epidemiological Studies-Depression Scale(CES-D)	Multi-centre, randomised, controlled trial<br><br>Statistic analyses: descriptive statistics; Pearson correlation coefficient, t-test and one-way ANOVA; Poisson log-linear regression; Wald Test; the Akiake Information Criterion (AIC)
23	Douglas, SL, Daly, BJ, Lipson, AR	2016	299 patient-caregiver dyads	- Demographic and clinical informations: interview<br>- HRQoL for patients: Functional Assessment of Cancer Therapy–General (FACT-G)<br>- Mood disturbance: Profile of Mood States–Short Form (POMS-SF)	Quasi-experimental, two-group<br><br>Statistical analysis: Pearson's correlation analysis; analysis of variance (RMANOVA; multiple linear regression

24	Johansen, S, Cvancarova, M, Ruland, C	2017	281 patient-caregiver dyads	- Demographic informations: questionnaire<br>- Comorbidity: Charlson comorbidity index<br>- Caregiver's burden: Caregiver Reaction Assessment (CRA),<br>- Fatigue: Lee Fatigue Scale (LFS)<br>- Sleep disturbance: General Sleep Disturbance Scale (GSDS)<br>- Depression: Center for Epidemiologic Studies Depression Scale (CES-D)<br>- Social support: Medical Outcomes Study Social Support Survey (MOS-SS)<br>- Self efficacy: Cancer Behavior Inventory (CBI) - patients; General Self Efficacy Scale (GSE) - caregivers<br>- Symptoms: Memorial Symptom Assessment Scale (MSAS)	Cross-sectional study<br><br>Statistical analysis: descriptive analysis; t-test; chi^2 tests
Methods of supportive intervention for patients, caregivers and health care roles involved in cancer diagnosis settings					
25	Lambert, SD, Duncan, LR, Ellis, J, et al.	2020	19 patients<br>18 caregivers<br>6 Healthcare professionals	For screening of participants:<br>- Physical Activity Readiness Questionnaire for Everyone (PAR-Q+)<br>- Physical Activity (PA)<br>- Physical Activity Readiness Medical Examination (Parmed-X)<br><br>Then: Semi-structured qualitative interviews	Longitudinal, multi-center, qualitative study: (thematic analysis)
26	Heckel, L, Fennell, KM, Orellana, L, et a	2018	108 caregivers	- Distress and impact of distress on daily life activity: Distress Thermometer (DT); interview<br>- Demographic characteristics: baseline questionnaire<br>- Utility survey: seven-item questionnaire	Multi-center, randomized, controlled trial<br><br>Statistic analyses: descriptive statistics; generalized linear models (GEE) with binomial distribution and logit link for binary variables; unstructured covariance matrix; models including double and triple interactions
27	Livingston, P, White , V, Hayman, J, et al	2006	100 patients	- Participants feelings, expectations, perspectives of received information: Computer-Assisted Telephone Interviewing (CATI)	Three amrs block randomized controlled trial<br><br>Thematic analysis and descriptive statistics
28	Livingston, PM, White, VM, Hayman, J, et al	2010	571 patients	- Cancer specific distress: modified version of a scale developed for breast cancer patients<br>- Anxiety and depression: Hospital Anxiety and Depression Scale (HADS)	- Three arms randomised controlled trial<br><br>Statistic analyses: random effects regression models

				- Optimism: single item<br>- Preference for involvement in medical decisions: Control Preference Scale (CPS)<br>- Social support: single item	
29	Hendrix, CC, Bailey, DE, Steinhauser, KE, et al	2016	138 patient-caregiver dyads	- Demographic data, functional status, well-being, self efficacy, preparedness: baseline questionnaires<br>- Self-efficacy for symptoms and stress management: Self-efficacy Scale for Cancer Caregivers<br>- Preparedness for caregiving: Preparedness for Caregiving scale, from Family Caregiving Inventory<br>- Anxiety: Profile of Mood States (POMS) subscale<br>- Depression: Center for Epidemiology Studies - Depression Scale (CES-D)<br>- Caregiver burden: Caregiver Reaction Assessment (CRA)<br>- Rapid Estimate of Adult Literacy in Medicine (REALM-R)<br>- Patient functional status: OARS Multidimensional Functional Assessment Questionnaire (OMFAQ)<br>- Well-being: Quality of Life in Chronic Illness (FACT-G)	Two-armed, randomized controlled trial<br><br>Statistic analysis: general linear models; unstructured covariance matrix; sequential regression method; Kaplan-Meier estimators and log-rank test statistic
30	Kleijn, G, Lissenberg-Witte, BI, Bohlmeijer, ET, et al	2020	64 caregivers	- Caregiver burden: Caregivers Reaction Assessment Scale (CRA)<br>- Anxiety and depression: hospital anxiety and depression scale (HADS)<br>- Posttraumatic growth: Post-traumatic Growth Inventory (PTGI)<br>- Demographic data: questionnaires	Randomized controlled trial<br><br>Statistical analyses: descriptive statistics; linear mixed models; effect size and independent samples t tests
31	Serrano Selva JP, Latorre Postigo JM, Ros Segura L, et al	2012	37 patients	- Psychiatric disorders: Mini-International Neuropsychiatric Interview (MINI)<br>- Depression: Geriatric Depression Scale (GDS)<br>- Expectancies: Beck Hopelessness Scale (BHS)<br>- Satisfaction: Life Satisfaction Index A (LSIA<br>- Quality of life: Quality of Life in Depression Scale (QLSD)<br>- Ability to retrieve memory: Autobiographical Memory Test (AMT)	Randomized trial<br><br>Statistical analyses: t-tests and chi-square; PROC-MIXED; analysis of variance
32	Moradi AR, Moshirpanahi S, Parhon H, et al.	2014	24 veterans	- Memory specificity: Autobiographical Memory Test (AMT)<br>- Severity of symptoms of posttraumatic intrusion, avoidance and hyperarousal: Impact of Event Scale-Revised (IES-R)<br>- Depression: Beck Depression Inventory-II (BDI-II)<br>- MEmory Specificity Training (MEST)	Pilot randomized controlled trial<br><br>Statistical analyses: descriptive statistics; t-test; mixed ANOVA

33	Raes F., Williams J. M. G., Hermans D	2009	10 patients	- Depression: Major Depression Questionnaire (MDQ); Beck Depression Inventory (BDI-II)<br>- Memory specificity: Autobiographical Memory Test (AMT); Sentence Completion for Events from the Past Test (SCEPT)<br>- Rumination on sadness: Leuven Adaptation of the Rumination on Sadness Scale (LARSS)<br>- Problem solving skills: Stress Anxiety Depression version of the Means-Ends Problem Solving task (SAD-MEPS)<br>- Negative attitudes about future: Beck Hopelessness Scale (BHS)<br>- Avoidance: Acceptance and Action Questionnaire (AAQ-II)	Preliminary evaluation of MEmory Specificity Training (MEST)<br><br>Statistical analyses: t-test; Kolmogorov-Smirnov and Shapiro-Wilk tests; ANOVA
34	Lozano-Lozano, M, Martín-Martín, L, Galiano-Castillo, N, et al	2020	80 patients	- Quality of Life: European Organization for Research and Treatment of Cancer Quality of Life Questionnaire Core 30 (EORT QLQ-C30) v3.0; breast cancer module Breast Cancer-Specific Quality of Life Questionnaire (EORT QLQ-BR23)<br>- Functional assessment:<br>- Disability: Disabilities of the Arm, Shoulder and Hand (DASH)<br>- active range of motion (AROM) of the shoulder plastic universal goniometer with the Norking and White approach<br>- Upper-body muscular strength: digital handgrip (TKK 5101 Grip-D)<br>- Body mass index, percentage fat mass, and bone mineral density: dual-energy X-ray absorptiometry	Assessor-blinded, randomized, controlled, parallel-group design<br><br>Statistical analysis: descriptive statistics; covariance analysis
35	Jenniches, I, Lemmen, C, Cwik, JC, et al.	2020	3484 patients with primary diagnosis of cancer	- Depression and anxiety: Hospital Anxiety and Depression Scale (German Version)<br>- Patients and public involvement: *ad hoc* questionnaires<br>- Appropriateness and feasibility of the stepped-care programme: written postal survey<br>- Satisfaction for patients: questionnaire and interviews<br>- Impact of acute cancer treatment, health and medical history and comorbidities on the effectiveness of psycho-oncological care and the changing use of healthcare services during the care programme: datasets analysis	- Regression Discontinuity Design (RDD), with control (HADS<15) vs intervention group (HADS>15)<br>- Evaluation process: mixed-methods design (qualitative and quantitative data), with Medical Research Council (MRC) framework and the consolidated framework for implementation research (CFIR)<br>- Dillman's 'Total Design Method for implementation of response rate
36	Johnson, S, Clayton, J,	2016	210 patient–caregiver	Compliance between patient's EOL wishes and the care provided:<br>- Demographic characteristics: Demographic questionnaire	Prospective, multisite, randomised control trial

	Butow, PN, et al		dyads	- Patient understanding of survival time: prognosis survey; iTool<br>- Patient/family/healthcare provider communication about EOL care: EOL communication with family and healthcare providers questionnaires<br>- Quality of Life: EQ-5D5L<br>- Preference for quantity or quality of life: Discrete choice experiment (DCE)<br>- Patient satisfaction with care: Satisfaction with care survey<br>- Costs of ACP: costs of care survey<br>- documentation of patient preferences for EOL care and concordance with care received at the EOL: Medical record review form<br>- Prevalence, timing and location of EOL care documents: medical records review form<br>- Place of death: interview<br>- Quality of EOL care: study specific 27-item tool; interview; Quality about EOL Communication (QOC)<br>- The impact of death on surviving family members: Impact of Events Scale (IES); Hospital Anxiety and Depression Scale (HADS)	Statistical analyses: $\chi 2$ tests; t-test; mixed models and generalised linear mixed models; descriptive statistics; Bland-Altman plots; Kaplan-Meier plots
37	Johnson, SB, Butow, PN, Bell, ML, et al	2018	210 patients	- Quality of EoL care: 27-item structured interview<br>- Anxiety, depression and quality of life: Hospital Anxiety and Depression Scale (HADS)<br>- Impact of death: Impact-of-Events Scale (IES)<br>- Patient–family and patient–healthcare provider communication about EoL care<br>- Patient and caregiver satisfaction with care: 5 questions survey<br>- Patient and family satisfaction with the ACP intervention: 9-item study-developed questionnaire<br>- Intervention fidelity: standardised form and coding protocol	Prospective multi-site randomised controlled trial with two parallel groups<br>Statistical analyses: chi-square and t-tests; mixed models; Fisher's exact test; Wilcoxon Rank Sum test; linear mixed models; logistic regression
38	Nottelmann, L, Jensen, LH, Vejlgaard, TB, et al	2019	132 patients<br>96 caregivers	Symptoms and QoL: European Organisation for Research and Treatment of Cancer Quality of Life Questionnaire (EORTC QLQ-C30)	N/A<br><br>Descriptive statistics

39	Greer, JA, Jacobs, JM, El-Jawahri, A, et al	2018	350 patients	- Sociodemographic and clinical characteristics: Eastern Cooperative Oncology Group (ECOG); Charlson comorbidity index (CCI)<br>- Coping strategies: Brief COPE;<br>- Health Insurance Portability and Accountability Act compliant Web-based survey tool<br>- Quality of Life: Functional Assessment of Cancer Therapy General (FACT-G)<br>- Depressive symptoms. Patient Health Questionnaire–9 (PHQ-9)	Nonblinded, randomized clinical trial<br><br>Statistical analysis: central tendency; linear regression models; Preacher and Hayes method; false discovery rate (FDR) control method
40	Quillen, LJ, Borstelmann, NA, Stanton, KE, et al.	2020	41 OSW	77-item survey (attitudes, practice behaviors, training experiences and needs, demographic characteristics, open comments)	Cross-sectional survey<br><br>Statistical analysis. descriptive statistics
41	Irwin, M, Dudley, W, Northouse, L, et al	2018	2055 oncology nurses	Online survey (nurses' practice and confidence in conducting caregiver assessment and intervention; nurses' knowledge of evidence-based interventions to address caregiver strain and burden	Pre-/post-test design<br><br>Statistical analysis: descriptive statistics; chi-square analysis; Text Analytics for Surveys, version 4.0.1; Akaike information criterion, Bayesian information criterion (BIC), sample size–adjusted BIC; Lo-Mendel-Rubin likelihood ratio test; Vermunt 3-step approach; Two-tailed tests
The QoL and well-being evaluation tools					
42	European Organization for Research and Treatment of Cancer	N/A	N/A	Quality of life questionnaires	N/A
43	Thompson, A, Turner, A.	2020	N/A	Comparison of the EQ-5D-3L and EQ-5D-5L: General Practice Patient Survey (GPPS)	N/A<br><br>Statistical analyses: Index of Multiple Deprivation (IMD); descriptive analysis; L1 statistic; Shannon–Weaver index (H'); Shannon's Evenness index (J');
44	Weitzner, MA, Jacobsen, PB,	1999	96 caregivers, 70 patients	- Impact of the patient's cancer illness on the QoL of the caregiver, impact on functioning: semi-structured interview	Three phases design: (1) item generation (2) item reduction and (3) validity and reliability testing.

	Wagner Jr, H, et al.			- Health state: Medical Outcomes Study Short Form-36 (SF-36)<br>- Depression: Beck Depression Inventory (BDI)<br>- Anxiety: State–Trait Anxiety Inventory (STAI)<br>- Burden: Caregiver Burden Scale (CBS)<br>- Performance status: Eastern Cooperative Oncology Group (ECOG) ;<br>- Performance Status Rating (PSR)<br>- Social support: Multidimensional Scale of Perceived Social Support (MSPSS)<br>- Desirability: Marlowe–Crowne Social Desirability Scale (MCSDS)<br>- Quality of life: Caregiver Quality of Life (CQOLC)	Statistical analyses: descriptive statistics, Pearson's correlation; Cronbach's α; analysis of variance
45	Yakar, HK, Pinar, R	2013	120 caregivers	- Quality of life: Caregiver Quality of Life Index-Cancer Scale (CQOLC); Medical Outcomes Study (MOS)<br>- Depression: Beck Depression Inventory (BDI),<br>- Health state: Short Form Health Survey (SF-36)<br>- Anxiety: State–Trait Anxiety Inventory (STAI)<br>- Social support: Multidimensional Scale of Perceived Social Support (MSPSS)	N/A<br><br>Statistical analyses: Internal consistency and test-retest stability; Known group method, convergent, and divergent validity
46	Sugiyama, I, Shoji, H, Igarashi, N, et al	2017	400 caregivers	Quality of Life: Japanese version of Caregiver Quality of Life Index-Cancer (CQOLC): internet survey	N/A<br><br>Statistical analyses: correlation analysis; factor analysis
47	Ando, S, Harata, M, Weitzner, MA, et al	2013	400 caregivers	Validation of the Japanese version of Caregiver Quality of Life Index-Cancer (CQOLC)<br>Quality of life: CQOLC and Short Form Health Survey (SF-36)	N/A<br><br>Statistical analysis: factor analysis; correlation analysis; cronbach' alpha
48	Chung, KJ, Kim, JJ, Lim, SH, et al	2010	153 patients	Quality of life after prostate cancer treatment: Expanded Prostate Cancer Index Composite (EPIC)	Translation, back-translation, and reconciliation.<br><br>Statistical analyses: test-retest correlation; Cronbach's alpha; factor analysis, interscale correlation, and correlation with Functional Assessment of Cancer Therapy-Prostate
49	Shin, DW, Shin, J, Kim, SY, et al.	2014	990 patient-caregiver dyads	- Perception of avoidance: Family Avoidance of Communication about Cancer (FACC) Scale<br>- Anxiety and depression: Hospital Anxiety and Depression Scale	Standard forward–backward translation process (for linguistic validation)

				- Quality of life: European Organization on Research and Treatment on Cancer Quality of Life Questionnaire core module-C30; Caregiver Quality of Life Scale	Statistical analysis: correlation analysis; descriptive analysis; analysis of covariance; Pearson's correlation coefficients
50	Siminoff, L A, Zyzanski, SJ, Rose, JH, et al	2008	190 lung cancer patients-caregivers dyads	- Validation of scale's results: semi-structured interview<br>- Depression: Center for Epidemiologic Studies Depression Scale (CES-D)<br>- Quality of life: Functional Assessment of Cancer Therapy -Lung (FACT-L) for patients and Medical Outcomes Study (MOS) short form for caregivers<br>- Family function: Family environment scale (FES); Family Relationship Index (FRI)	Validation of the Cancer communication Asessment Tool for patients and families (CCAT-PF) through items reduction, by: one-sample t-test; multiple correlation analysis and Kendall's coefficient of correlation; Kappa statistic; backward elimination regression analyses.<br>Psychometric properties tested reliability (Cronbach's internal consistency reliability and test re-test) and concurrent validity (Pearson correlation statistic)
51	O'Donnell, E, D'Alton, P, O'Malley, C, et al.	2013	228 patients	Emotional distress: Distress Thermometer	Four-phases intervention to healthcare professionals and patients: gaining staff perspectives; staff training; introducing the Distress Thermometer; assessing patients and staffs DT experience
52	Cella, DF, Tulsky, DS, Gray, G, et al	1993	854 patients, 15 oncology specialists	Quality of life: interviews for the generation of the FACT-G	Five-phase validation process.<br><br>Statistical analyses: t-test, Cronbach α; Pearson correlation
53	Mah, K, Swami, N, Le, LW, et al	2020	461 patients	- Quality of Life: Functional Assessment of Cancer Therapy General (FACT-G7)<br>- Symptoms' severity: Edmonton Symptom Assessment System (ESAS)<br>- End-of-life Quality of Life: Quality of Life at the End of Life - Cancer (QUAL-EC) scale<br>- Perceived problems with medical communication and interaction: Cancer Rehabilitation Evaluation System-Medical Interaction Subscale (CARESMIS)<br>- Patients' satisfaction with cancer care: FAMCARE-Patient Scale (FAMCARE-P16)<br>- Performance status: Eastern Cooperative Oncology Group (ECOG)	Validation of the 7-Item Functional Assessment of Cancer Therapy-General (FACT-G7) by: randomized controlled trial and statistical analyses: t-test; consistency - with Cronbach α; single-factor structure, confirmatory factor analysis (CFA); weighted least-squares estimation; convergent and discriminant validity - with Pearson correlations; criterion validity - with 1-way analysis of variance

Oncological diagnosis repercussion					
54	Callahan, C, Brintzenhofeszoc, K	2015	90 patients	- Financial Quality of Life: Socioeconomic Well-Being Scale (SWBS)<br>- Housing instability: set of six questions<br>- Personal control: Multidimensional Health Locus of Control Scale<br>- Demographic informations<br>- Income and financial stress: response to dollar categories; three questions from the Profile of Adaptation to Life (PAL-M)<br>- Presence of health insurance and health insurance adequacy: set of questions<br>- Perceived barriers to care: modified questions from the basic need satisfaction subscale of the Quality of Life Questionnaire (QLQ)<br>- Social support: social support subscale of the Quality of Life Questionnaire (QLQ)<br>- Patient's experience with cancer: composite index<br>- Perceived ability to participate meaningfully in treatment: Adherence Determinants Questionnaire (ADQ)	Cross-sectional research<br><br>Statistical analysis: regression analysis
55	Boele, FW, Meads, D, Jansen, F, et al	2020	90 patients and 45 caregivers	- Costs of healthcare utilization: Trimbos/iMTA questionnaire for Costs associated with Psychiatric Illness (TIC-P), incorporating the Short-Form Health and Labor Questionnaire (SF-HLQ)<br>- Depression: Epidemiological Studies-Depression Scale (CES-D)<br>- Fatigue: Checklist Individual Strength (CIS)<br>- Cognitive complaints: MOS cognitive functioning scale<br>- Symptoms (only patients): EORTC Brain Cancer Module	Multicenter randomized trial<br><br>Statistical analyses: descriptive statistics; generalized linear regression models ; multivariate model with backward selection
56	Short, PF, Vasey, JJ, Tunceli, K.	2005	1763 patients	- Clinical details: medical records<br>- Employment data: interviews	N/A<br><br>Statistical analysis: logit analysis (life-table estimates; Log-rank tests; Multivariate analyses; Ordered logit; Standard dichotomous logit; Wald chi-square)
57	Short, PF, Vasey, JJ, Moran, JR	2008	405 cancer survivors<br>94 newly diagnosed	- Employment and specifics (health insurance, geographic differences, sociodemographic characteristics): interviews<br>- Clinical details: medical records	Longitudinal study<br><br>Statistical analyses: Probit/Tobit regressions; covariance; kernel matching and k-nearest neighbor matching; "max–min'' criteria and "trimming"

			5 patients with no new cancer		
58	Mols, F, Thong, MS, Vreugdenhil, G, et al	2009	1511 long term cancer survivors	- Clinical data: Eindhoven Cancer Registry (ECR)<br>- Comorbidity: Charlson comorbidity index<br>- Demographic data and employment characteristics: questionnaire<br>- health-related quality of life (HRQL): Short Form Health Survey (SF36)<br>- Disease-specific HRQL: Quality of life-Cancer Survivors (QOL-CS)	Population-based cross-sectional survey<br><br>Statistical analyses: chi-square test; analysis of variance; logistic regression; Pearson correlation
59	Law, C, Brewer, K, Brown, C, et al.	2021	473 patients	- Medical data: medical database<br>- Surgical outcomes: medical database<br>- Socio-demographic data and employment data: survey;: Eastern Cooperative Oncology Group Scale	Randomized trial<br><br>Statistical analyses: descriptive statistics; univariate analysis (Pearson chi-square ($\chi 2$) tests and t-test); multivariate logistic regression models; ODD's ratio; logistic regression model;
60	Hall, ET, Sridhar, D, Singhal, S, et al	2020	29 participant (11 patients, 7 caregivers, 11 health professionals)	- Attitudes about cancer care and the impact on their life, including attitudes surrounding logistics and processes of cancer care: semi-structured interviews<br>- Demographic data	Coding of text (inductive methods)
61	Hwang, IC, Yun, YH, Kim, YW, et al.	2014	374 patients	- Demographic and clinical data: questionnaire<br>- Fatigue: Brief Fatigue Inventory (BFI)<br>- Depression: Beck Depression Inventory (BDI)<br>- Health-related Quality of Life: European Organization for Research and Treatment of Cancer (QLQ-C30) questionnaire + gastric module QLQ-STO22<br>- Performance status: Eastern Cooperative Oncology Group (ECOG)	Cross-sectional study<br><br>Statistical analysis: multivariate logistic regression models
62	Miller, KD, Nogueira, L, Mariotto, AB, et al	2019	N/A	- Cancer survivor prevalence and mortality data: Prevalence Incidence Approach Model<br>- Case estimates: Spatiotemporal model based on incidence data<br>- Stage at diagnosis: American Joint Committee on Cancer (AJCC) staging system and Surveillance, Epidemiology, and End Results (SEER) program<br>- Survival information: Surveillance, Epidemiology, and End Results (SEER) program	Statistical estimations

				- Initial treatment data: National Cancer Data Base (NCDB)	
63	Takvorian, SU, Balogh, E, Nass, S, et al.	2020	N/A	- Considered factors: demographic and workforce trends; increasing complexity of cancer care; increasing administrative burden; clinical burnout.<br>Considered strategies: Strategies focusing on patients; strategies focusing on informal caregivers; strategies focusing on clinicians.	Commentary about factors contributing to a stressed oncology workforce and strategies to build an effective and resilient oncology careforce
Health value					
64	Turchi, GP, Vendramini, A.	2016	N/A	N/A	N/A
65	Lubberding, S, van Uden-Kraan, CF, Te Velde, EA, et al	2015	15 patients	Interviews:<br>- Unmet needs during regular follow-up care<br>- Acceptability and preferences towards an eHealth application	Qualitative approach: coding of text
66	Mooney, KH, Beck, SL, Wong, B, et al.	2017	358 patients	- Demographic data: clinical records<br>- Presence and severity of 11 symptoms (fatigue, trouble sleeping, nausea and vomiting, pain, numbness or tingling, feeling blue or down, feeling nervous or anxious, distress over appearance, diarrhea, sore mouth, and trouble thinking or concentrating): automated system for remotely monitoring chemotherapy symptoms	Longitudinal randomized controlled trial<br><br>Statistical analyses: descriptive statistics; power analysis via longitudinal mixed modeling; Pearson Chi-square and t-test; mixed effects linear models
67	Pfeifer, MP, Keeney, C, Bumpous, J, et al.	2015	80 patients	Interviews:<br>- Quality of Life: Functional Assessment of Cancer Therapy - Head&Neck Scale (FACT-HN)<br>- Symptom burden: Memorial Symptom Assessment Scale (MSAS).	Parallel two-group randomized clinical trial<br><br>Statistical analyses: priori power calculations; descriptive statistics;
